# Cancer‐associated fibroblasts promote tumor progression by lncRNA‐mediated RUNX2/GDF10 signaling in oral squamous cell carcinoma

**DOI:** 10.1002/1878-0261.12935

**Published:** 2021-06-10

**Authors:** Dongya Zhang, Yuxian Song, Dan Li, Xinghan Liu, Yuchen Pan, Liang Ding, Guoping Shi, Yong Wang, Yanhong Ni, Yayi Hou

**Affiliations:** ^1^ The State Key Laboratory of Pharmaceutical Biotechnology and Central Laboratory of Stomatology Nanjing of Stomatological Hospital Division of Immunology Medical School Nanjing University China; ^2^ State Key Laboratory of Analytical Chemistry for Life Science Medical School Nanjing University Nanjing China; ^3^ Jiangsu Key Laboratory of Molecular Medicine Medical School Nanjing University Nanjing China

**Keywords:** cancer‐associated fibroblasts, lncRNA, transformation, tumor progression

## Abstract

Cancer‐associated fibroblasts (CAF) are the most abundant stromal cells in tumor and exert a pro‐tumoral effect in cancer progression. Numerous evidence shows long non‐coding RNA (lncRNA) abnormally regulates gene expression in various cancers. However, little is known about the role of lncRNA in the interaction between CAF and cancer cells. Here, we first identify an uncharacterized lncRNA, LOC100506114, which is significantly upregulated in CAF and is involved in the functional transformation of normal fibroblasts (NF) and CAF. Expression of LOC100506114 enhances the expression of fibroblast activation protein alpha and α‐smooth muscle actin in NF and promotes malignant characteristics of NF and CAF *in vivo* and *in vitro*. The profile of gene co‐expression analysis shows that growth differentiation factor 10 (GDF10) is positively correlated with the expression of LOC100506114. CAF promote stromal fibroblast activation and the proliferation and migration of tumor cells by secreting GDF10. Our data demonstrate that lncRNA plays a critical role in the interplay of stromal fibroblasts and tumor cells in oral squamous cell carcinoma.

## Graphical abstract

Abnormal expression of lncRNA LOC100506114 in oral squamous cell carcinoma (OSCC) induced normal fibroblasts (NF) to transform into CAF by upregulating growth differentiation factor 10 (GDF10) expression and secretion. The interaction of GDF10 with the transcription factor RUNX family transcription factor 2 further enhanced the growth and migration of OSCC tumor cells by activating the TGFβRI/Smad3/ERK pathway. Our results demonstrate that lncRNA play an important role in the functional transformation of human NF into CAF and promote OSCC tumorigenesis.

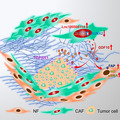

**Abbreviations**
CAF: cancer‐associated fibroblasts
CM: conditioned medium
FAP: fibroblast activation protein alpha
FISH: fluorescence *in situ* hybridization
FSP1: fibroblast specific protein 1
GDF10: growth differentiation factor 10
lncRNA: long non‐coding RNA
NF: normal fibroblast
OSCC: oral squamous cell carcinoma
RNA‐seq: RNA sequencing
RUNX2: RUNX family transcription factor 2
si‐: small interfering
TGF‐β: transforming growth factor‐β
α‐SMA: α‐smooth muscle actin

## 1 Introduction

Oral squamous cell carcinoma (OSCC) is the sixth most common tumor in the world and accounts for more than half of patients with oral cancer [1, 2]. Despite the great advances of cancer therapy, local recurrence and regional lymph node metastasis remain common in most patients with OSCC [3]. It is well known that the interplay of tumor stroma and tumor cells exerts a profound effect on tumor development [4]. In particular, cancer‐associated fibroblasts (CAF) have been identified as an important component in tumor stroma that play a prominent role in tumor progression in a paracrine manner [5, 6]. In terms of malignant properties, compared with resting fibroblasts, CAF promote tumorigenesis, metastasis, recurrence and therapeutic resistance via various soluble factors [7, 8, 9]. There is evidence that α‐smooth muscle actin (α‐SMA)‐positive CAF are significantly associated with poor prognosis in patients with OSCC [10]. Numerous observations reveal that CAF promote OSCC progress [11, 12, 13]. In addition, enriched CAF have a considerable influence on invasion and metastasis of tumor cells in OSCC [14]. We have also demonstrated that CAF enhance tumor growth and chemotherapy resistance in OSCC [15, 16]. However, as one of the sources of CAF, little is known about the mechanism of how normal fibroblasts (NF) transform into CAF in OSCC.

Long non‐coding RNA (lncRNA) is a category of transcripts of more than 200 nucleotides in length, lacking a protein‐coding function [17]. Currently, it is believed that the aberrant expression of lncRNA is associated with various diseases, such as autoimmunity, amyotrophic lateral sclerosis and tumors [18, 19, 20]. The lncRNA regulates the transcription and expression of genes by interacting with biological macromolecules such as DNA, proteins and RNA via four mechanisms: signaling molecules, decoys, guides and molecular scaffolds [21]. It has been reported that the elevated expression of CXCL14 in CAF mediated upregulation of LINC00092 in ovarian cancer cells and caused metastatic progression [22]. Emerging evidence suggests that the abnormal expression of lncRNA in tumor stroma regulates tumor progression by exosome transportation [23, 24]. We have previously shown that the exosomal lncRNA H19 secreted by CAF promotes the stemness and drug resistance of colorectal cancer cells [25]. Furthermore, NF remodeled by the tumor microenvironment were reported to transform into pro‐tumorigenic CAF by microRNA. There is some evidence supporting the involvement of lncRNA in the transformation of NF into CAF. We have also previously shown differential expression of lncRNA in NF and CAF [26]. However, the role of lncRNA that plays in resting fibroblast transformation into CAF in tumors is unknown. Therefore, it is important to reveal further the underlying mechanism of stromal fibroblast transformation in order to explore new methods for targeting tumor stroma therapy in the future.

In this study, paired mesenchymal fibroblasts isolated from clinical OSCC patients demonstrated that abnormal expression of lncRNA in fibroblasts sustained the growth and invasion of OSCC cells in a paracrine manner by CAF. Most importantly, our findings revealed that LOC100506114 was involved in reprogramming NF into CAF in OSCC. Mechanistically, upregulation of growth differentiation factor 10 (GDF10) levels by lncRNA LOC100506114 binding with RUNX family transcription factor 2 (RUNX2), promoted fibroblast activation and promoted OSCC cell proliferation and migration via activation of the TGFβR1/Smad3/ERK pathway of OSCC cells. These results indicate that lncRNA plays an important role in the formation of CAF.

## 2 Materials and methods

### 2.1 Tissue specimens

Fourteen paired OSCC specimens and adjacent normal tissues were obtained from Nanjing Stomatology Hospital. Five pairs were used for RNA sequencing. The use of OSCC specimens for this study was approved by the Research Ethics Committee of Nanjing Stomatology Hospital affiliated to Nanjing University. All subjects diagnosed with other diseases were excluded from this study. None of the patients underwent preoperative chemotherapy or radiotherapy. In this study, all OSCC tissues were evaluated by pathologists according to WHO classifications and the International Cancer Control (UICC) tumor staging system.

### 2.2 Isolation and primary culture of fibroblasts

Oral squamous cell carcinoma specimens and adjacent normal tissues were harvested within 30 min after surgical resection. Harvested tissues were placed in Dulbecco’s modified Eagle’s medium (DMEM)/F‐12 supplemented with 10% FBS and antibiotics (Life Technologies, Carlsbad, CA, USA) for immediate transportation on ice to the laboratory. NF and CAF were isolated from tissues by a combination of mechanical and enzymatic methods. Details of tissue preparation have been described previously [26]. The sterile, fresh OSCC tissues and their corresponding normal tissues were washed in PBS and antibiotics, and epithelial and adipose tissues were eliminated. The specimens were sliced into small pieces and digested in an enzyme mixture (collagenase, neutral protease, hyaluronidase) for 30 min. The remaining small tissues were incubated in DMEM/F12 medium with 20% FBS at 37 °C. Every 2–3 days, the medium was replaced and the epithelial cells were removed via trypsinization. The remaining cells were fibroblasts. For the identification of NF and CAF, we detected the expression of α‐SMA and fibroblast activation protein alpha (FAP) among the identified markers of CAF by immunofluorescence following previous studies. The spectral cytokeratin PanCK was used for the epithelial cells.

### 2.3 RNA sequencing

We performed RNA sequencing (RNA‐seq) with the help of Novel Bioinformatics Co., Ltd (Shanghai, China). Total RNA extraction, RNA‐seq and bioinformatics data analysis were performed by Novel Bioinformatics Co., Ltd. Details of the RNA‐seq and bioinformatics data analysis have been described previously [26].

### 2.4 Collection of conditioned media

Cells were grown in 10‐cm flasks until they were 70–90% confluent, washed with serum‐free media (×3) and PBS (×3), and then incubated in serum‐free media for a further 48 h. The conditioned media (CM) was centrifuged at 800 ***g*** for 5 min to remove dead cells and stored at −20 °C. The viable attached cells were trypsinized and counted. The CM was normalized with 0.5 × 10^6^ fibroblasts.

### 2.5 Colony formation assay

Oral squamous cell carcinoma cell lines were harvested 24 h before being treated with conditioned medium from NF or CAF by trypsinization, and then incubated for 2 weeks after seeding into 6‐well plates (2000 cells per well). Visible colonies were fixed with methanol and stained with 0.1% crystal violet. Colonies with more than 50 cells were counted.

### 2.6 Wound healing assay

Oral squamous cell carcinoma cell lines were harvested by trypsinization and seeded into 6‐well plates (8 × 10^5^/well) for 24 h before treatment. Cells were drawn using a 200‐µL tip until 90% confluency was achieved. The unattached cells were washed in PBS and incubated after treatment with conditioned medium from NF or CAF, as well as GDF10 followed by a photograph at the time indicated.

### 2.7 Cell proliferation assay

HSC3, OSCC3 and SCC4 were seeded at the density of 5000 cells per well in a 96‐well plate (100 µL per well) for 24 h after treatment with exogenous GDF10, with conditioned medium from NF or CAF. Cell viability was determined for 48 h using a Cell Counting Kit‐8 (CCK‐8; Dojindo Laboratories, Kumamoto, Japan) according to the manufacturer’s instructions, and the absorbance of each well was measured at 450 nm with a microtiter plate reader (Synergy HT, BioTek, Winooski, VT, USA). Cell viability was calculated as the ratio of treated to untreated cells.

### 2.8 Flow cytometry assay

Freshly removed spleens were mechanically disaggregated and tumor tissues from mouse xenograft model were mechanically minced into small pieces and digested in a 1‐mL mixture of 300 U·mL^−1^ collagenase IV (Roche, Basle, Switzerland), 300 U·mL^−1^ collagenase type I (Sigma, St. Louis, MO, USA) and 0.4 mg·mL^−1^ DNase I in RPMI 1640 medium at 37 °C for 1 h. After the incubation, the cell suspensions were filtered through a 70‐μm mesh and then washed with complete RPMI medium prior to immunostaining. Peripheral blood mononuclear cells (PBMC) from mice were first isolated by mouse PBMC separation solution kit (Hao Yang Biological Manufacturers, Tianjin, China) for detecting myeloid‐derived immunosuppressive cells (MDSC). The single‐cell suspensions isolated from the spleens and tumor were first incubated with Fc‐blocker anti‐CD16/32 antibody (dilution 1 : 20; Miltenyi Biotec, Bergisch Gladbach, Germany) for 15 min, followed by staining after pre‐incubation with anti‐CD45‐PerCP (BioLegend, San Diego, CA, USA), anti‐CD11b‐APC (1 μL per test) and anti‐GR‐1‐PE (0.3 μL per test) for 30 min at 4 °C in the dark. Cells were then washed with buffer to remove the excess stains and analyzed by FACS (Becton Dickinson, San Diego, CA, USA).

### 2.9 Immunofluorescence

Normal fibroblasts, CAF and SCC4 cells were seeded on coverslips in a 24‐well plate and cultured overnight. Subsequently, cells were fixed in 4% paraformaldehyde, permeabilized in 0.2% Triton X‐100 at room temperature, and then incubated with primary antibody anti‐α‐SMA antibody (Sigma), anti‐FAP antibody (Abcam, Cambridge, MA, USA) and anti‐PanCK antibody (Life Technologies) overnight at 4 °C after 5% BSA blocking for 1 h. After incubation for 2 h at room temperature with secondary antibody, the coverslips were counterstained with 0.2 mg·mL^−1^ DAPI, followed by washing in PBS, and sealed with nail polish and observed under an FV3000 confocal microscope (Olympus, Tokyo, Japan). Tissue immunofluorescence was performed as described for cell immunofluorescence.

### 2.10 Immunohistochemistry and FISH

Immunohistochemistry was performed as described in our previous study [27]. Slides were stained for the primary antibodies α‐SMA (Sigma, 1 : 40), PanCK (Life Technologies, 1 : 40) and GDF10 ( 1 : 200; R&D, Minneapolis, MN, USA). The expression of LOC100506114 in cells and tissues was determined by Cy3‐labeled LOC100506114 and fluorescence *in situ* hybridization (FISH) Kit (RiboBio, Guangzhou, China) according to the manufacturer’s instructions.

### 2.11 Western blot analysis

Cells were lysed in buffer containing 50 mmol·L^−1^ Tris/HCl, pH 8.0, 150 mmol·L^−1^ NaCl, 0.02% NaN_3_, 0.1% SDS, 100 mg·L^−1^ phenylmethylsulfonylfluoride, 1 mg·L^−1^ aprotinin and 1% Triton. Cell extract was separated by SDS/PAGE and transferred onto PVDF membranes. The membranes were blocked for 1 h in TBST (10 mmol·L^−1^ Tris/HCl, pH 7.4, 150 mmol·L^−1^ NaCl, 0.05% Tween‐20) containing 5% BSA, incubated with the primary antibodies anti‐E‐cadherin antibody (Proteintech), anti‐N‐cadherin antibody (Proteintech), anti‐vimentin antibody (Proteintech, Rosemont, IL, USA), anti‐α‐SMA antibody (Sigma), anti‐FAP antibody (Abcam), anti‐GDF10 antibody (R&D) at 4 °C overnight, followed by incubation with secondary antibodies. Bands were visualized with an enhanced chemiluminescence reaction (Millipore Corp., Billerica, MA, USA). GAPDH was used as the loading control. Protein bands were captured and analyzed using lane 1D software (Sage Creation Science Co., Beijing, China).

### 2.12 Lentivirus vectors

The GFP‐labeled lentivirus‐mediated overexpression vector containing LOC100506114 (Lv‐LOC100506114) was used stably to overexpress LOC100506114 in NF, with Lv‐ctrl as the matched control (GeneChem, Shanghai, China) according to the manufacturer’s instructions. The GFP‐labeled lentivirus vector expressing small interfering (si)‐LOC100506114 (Lv‐si‐LOC100506114) was used to knockdown LOC100506114 expression stably in CAF (Abm, Zhenjiang, China). The cells were treated with puromycin (5 μg·mL^−1^) for 2 weeks to establish stable cell lines.

### 2.13 Cell transfection

The siRNA LOC100506114 (si‐LOC100506114) and GDF10 (si‐GDF10) were obtained from GenePharma (Shanghai, China). The plasmid pcDNA3.1‐LOC100506114 used for overexpression of LOC100506114 was purchased from GenePharma. The si‐LOC100506114, si‐GDF10 or si‐NC was transfected into CAF. Cells were grown on 6‐well plates to 60% confluence and transfected using Lipofectamine 2000 (Life Technologies) according to the manufacturer’s instructions; the final concentration of siRNA was 60 nm. Cells were harvested for qPCR or western blot analysis 48 h after transfection. The siRNA sequences used in this study are provided in Table S1.

### 2.14 Reverse transcription and real‐time quantitative PCR analysis

Total RNA was isolated from cells or tissues using TRIzol reagent (Life Technologies) according to the manufacturer’s instructions. Reversed transcription for cDNA was carried out with the Reverse Transcription System at 42 °C for 10 min, 95 °C for 5 min and 4 °C for 5 min. The SYBR Green PCR Master Mix (Bio‐Rad, Hercules, CA, USA) was performed for quantitative real‐time PCR according to the manufacturer’s instructions. The qPCR assays were carried out on a StepOnePlus (Applied Biosystems, CA, USA) and data were collected with this instrument. The relative gene expression was calculated with the 2**^−∆∆^** ^CT^ formula, and the results were normalized to GAPDH. This method has been described previously [28]. All primer sequences are shown in Table S1.

### 2.15 Mouse xenograft experiments

Four‐ to six‐week‐old female BALB/c‐Foxn1nu/Nju nude mice were purchased from the Model Animal Research Center of Nanjing University (Nanjing, China) and raised in pathogen‐free housing conditions in a 12‐h light and dark cycle. All experiments were conducted in accordance with institutional guidelines for animal care based on the Guide for the Animal Care Committee at Nanjing University. HSC3 cells (1 × 10^6^) were injected subcutaneously into the right flank of each animal with NF or CAF mixed with Matrigel (Gibco, NY, USA) (0.5 × 10^6^). The tumor growth and mice weight were determined every 3 days for 1 week after the injection. Peripheral blood was collected from the medial canthus vein under anesthesia following the 4‐week injection. The tumor and spleen tissues were harvested from mice after sacrifice by anesthesia. The tumor tissues were paraffin‐embedded and serial sections were histologically examined with immunochemistry.

### 2.16 Statistics

Statistical analysis was performed using graphpad prism (San Diego, CA, USA). Two‐tailed Student *t*‐tests were used for comparison of experimental groups. Statistical significance was defined as ≥95% confidence interval or *P* < 0.05. The experiments presented are representative of three different repetitions. Data are presented as mean ± standard deviation.

Additional details can be found in Supporting Information.

## 3 Results

### 3.1 Upregulated expression of lncRNA LOC100506114 in CAF from OSCC patients

We isolated fibroblasts from adjacent tissues and tumor tissues of OSCC patients for later study. Under light microscopy, we observed that the fibroblasts separated from tumor tissues were larger and slender, with more branches and faster growth (Fig. S1A). In contrast to the fibroblasts from adjacent tissues of the same OSSC patients, a higher expression of FAP and α‐SMA was detected in fibroblasts from tumor tissues (Fig. S1B,C), whereas the epithelial cell marker PanCK was only expressed in tumor cells. There were a greater number of α‐SMA‐positive fibroblasts around the tumor cells in tumor tissue (Fig. S1D). The results are consistent with the well‐known characteristics of CAF [29, 30].

**Fig. 1:**
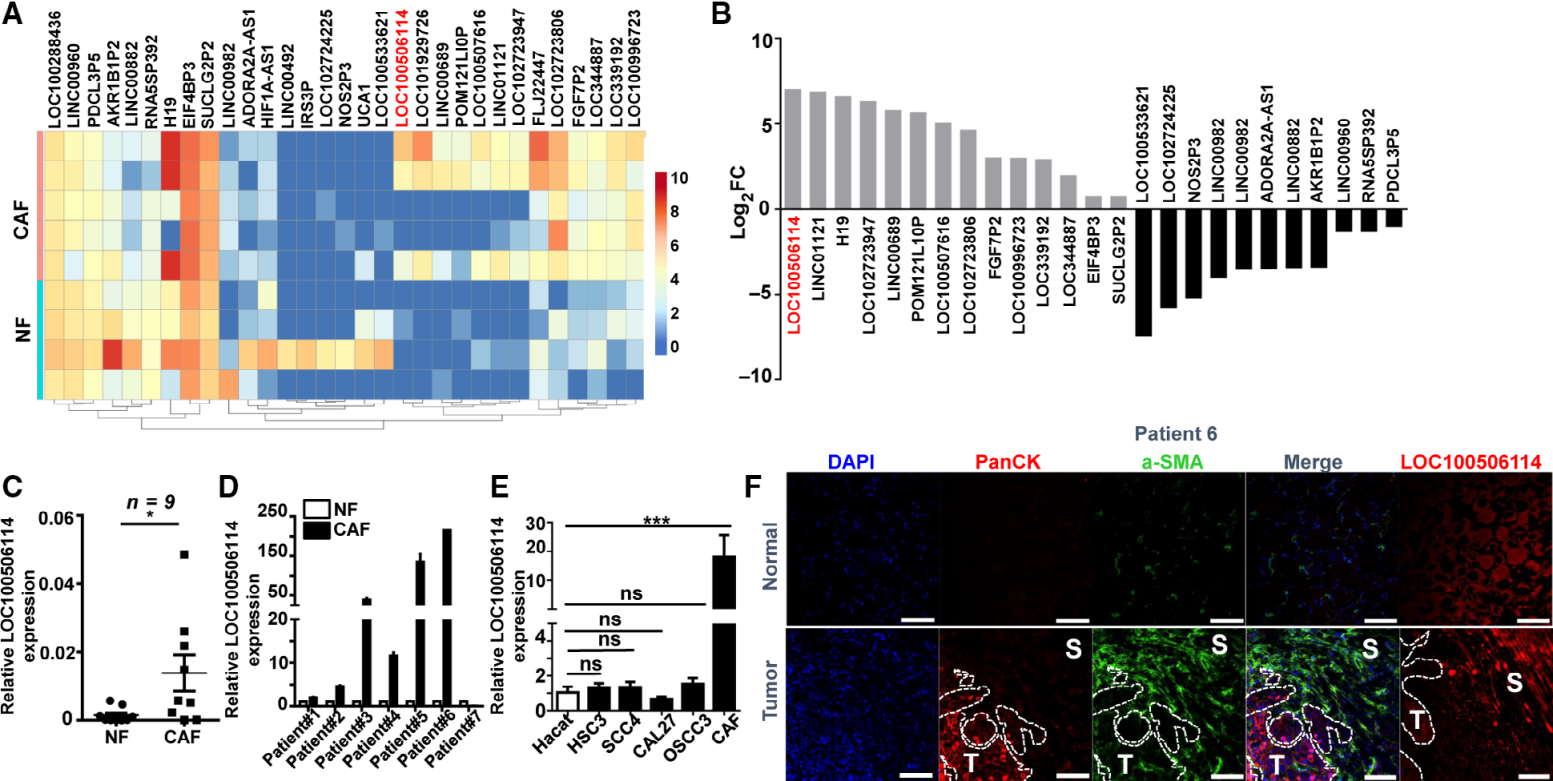
LOC100506114 is highly expressed in CAF. (A) The heat map of differential expression of lncRNA in NF and CAF. (B) The highest fold change of LOC100506114 in the expression analysis of lncRNA (A). (C) The expression of LOC100506114 in nine patient‐derived NF and CAF. (D) The expression of LOC100506114 in seven paired NF and CAFs. (E) The expression of LOC100506114 in HSC3, SCC4, OSCC3, CAL27 and human normal keratinocytes HaCaT and CAF. (F) The expression of LOC100506114 at the tissue level. LOC100506114 is primarily expressed in tumor stroma. T, tumor nest; S, stroma. Scale bars: 100 μm. All data are presented as mean ± SD. *P < 0.05, ***P < 0.001. Representative data from three independent experiments.

To investigate how the interstitial fibroblasts acquire the properties that promote the progression of malignant tumors, we performed the whole‐genome transcriptome profiling of five paired NF and CAF from OSCC patients by RNA‐seq. For bioinformatics analysis, we applied an EB‐seq algorithm to filter the differentially expressed genes among NF and CAF. The heat map provided a visual representation of the significant expression of lncRNA between NF and CAF, screened with the fold change of more than two and a false discovery rate of < 0.05 (Figs 1A,B, and S4). The fold change of lncRNA LOC100506114 was highest among lncRNA that changed between NF and CAF. To validate the results, we determined the expression of LOC100506114 in NF and CAF from nine different OSCC patients using qPCR, and randomly selected seven of them to determine the expression of LOC100506114 in pairs. Consistent with the result of RNA‐seq, the expression of LOC100506114 was obviously higher in CAF than in NF (Fig. 1C,D). Compared with four human OSCC cell lines and human immortalized keratinocyte HaCaT, the expression of LOC100506114 was significantly upregulated in CAF (Fig. 1E). The results of tissue immunofluorescence and RNA FISH showed that LOC100506114 was expressed in the tumor stroma site and cell nucleus, whereas the tumor nest site was not expressed (Fig. 1F). These results indicate that lncRNA LOC100506114 is involved in CAF function.

### 3.2 LOC100506114 regulates the expression of CAF phenotypic markers

**Fig. 2:**
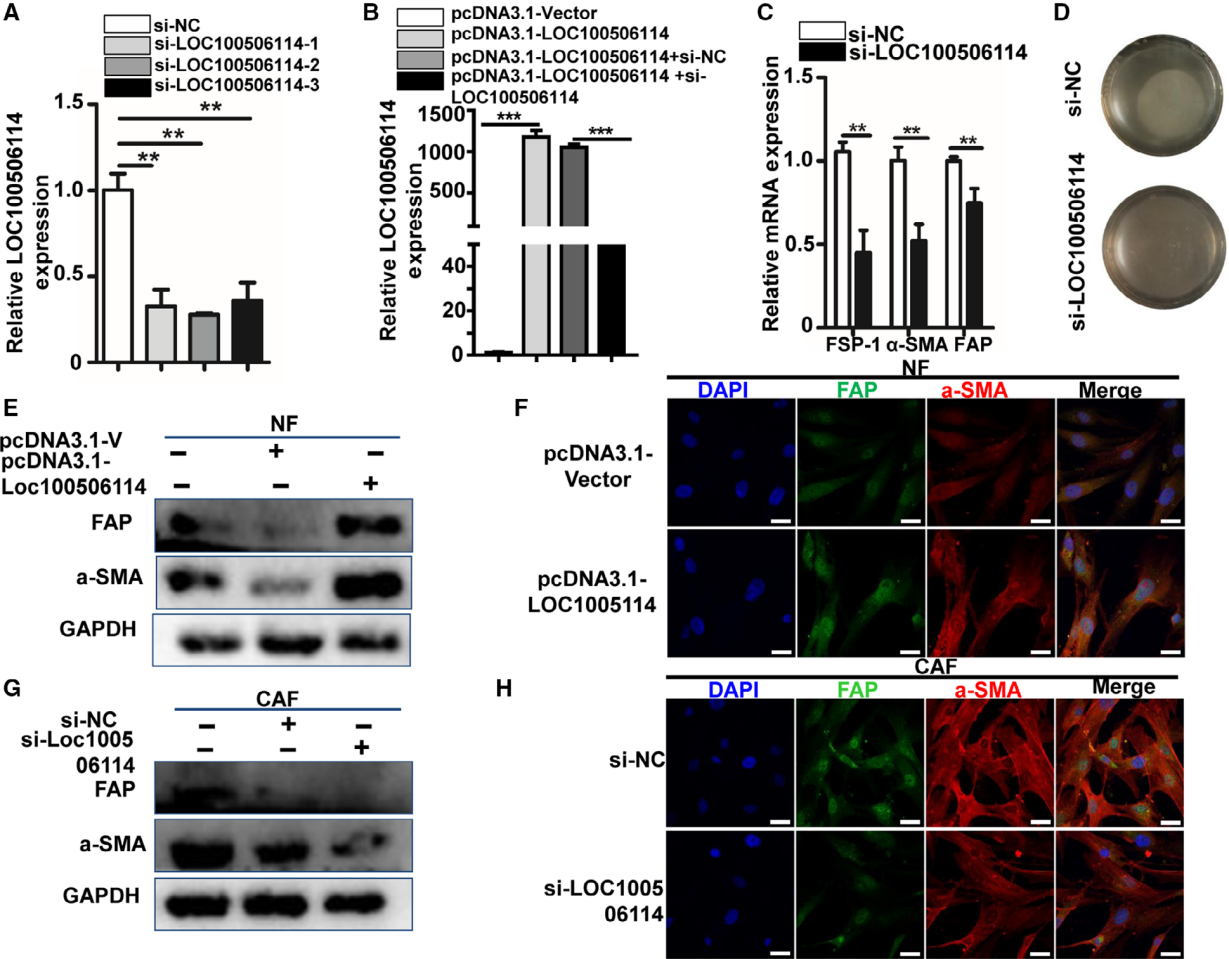
LOC100506114 regulates the expression of FAP and α‐SMA. (A) The expression of LOC100506114 was significantly inhibited in CAF transfected with si‐LOC100506114. (B) The expression of LOC100506114 in NF transfected with pcDNA3.1‐Vector or pcDNA3.1‐LOC100506114, or co‐transfected with si‐NC and pcDNA3.1‐LOC100506114 or si‐LOC100506114 and pcDNA3.1‐LOC100506114. NF transfected with pcDNA3.1‐Vector were used as control. (C) The expression of FSP‐1, α‐SMA and FAP was downregulated in CAF transfected with si‐LOC100506114. (D) The gel contractile ability of CAF transfected with siRNA‐LOC100506114 was weaker than that of the control group. (E) The expression of FAP and α‐SMA was detected by western blot in NF transfected with pcDNA3.1‐Vector or pcDNA3.1‐LOC100506114. (F) The expression of FAP and α‐SMA was detected by immunofluorescence in NF transfected with pcDNA3.1‐Vector or pcDNA3.1‐LOC100506114. Scale bars: 50 μm. (G) Western blots showing FAP and α‐SMA expression in CAF transfected with si‐NC or si‐LOC100506114. (H) The expression of FAP and α‐SMA was detected by immunofluorescence in CAF transfected with si‐NC or si‐LOC100506114. Scale bars: 50 μm. All data are presented as mean ± SD. **P < 0.01, ***P < 0.001. Representative data from three independent experiments.

To certify further the role of LOC100506114 in the functional transformation of CAF, we knocked down the expression of lncRNA LOC100506114 in CAF and overexpressed LOC100506114 in NF. The expression of LOC100506114 in CAF was successfully knocked down by si‐LOC100506114, but not in the si‐NC‐control group (Fig. 2A). Similarly, the expression of LOC100506114 in pcDNA3.1‐LOC100506114‐NF was significantly higher than that of NF transfected with the empty plasmid, whereas NF transfected with pcDNA3.1‐LOC100506114 and si‐LOC100506114 were clearly restored (Fig. 2B). Knockdown of LOC100506114 in CAF also markedly reduced the expression of fibroblast specific protein 1 (FSP‐1), FAP and α‐SMA at the RNA level (Fig. 2C). The downregulated expression of LOC100506114 inhibited the gel contraction ability of CAF (Fig. 2D). Additionally, overexpression of LOC100506114 promoted the expression of FAP and α‐SMA in NF (Fig. 2E,F), whereas the downregulated expression of LOC100506114 inhibited the expression of FAP and α‐SMA in CAF (Fig. 2G,H). These results reveal that LOC100506114 plays a significant role in the functional transformation of NF and CAF.

### 3.3 LOC100506114‐reprogrammed fibroblasts promote OSCC cell proliferation and migration *in vitro*

**Fig. 3:**
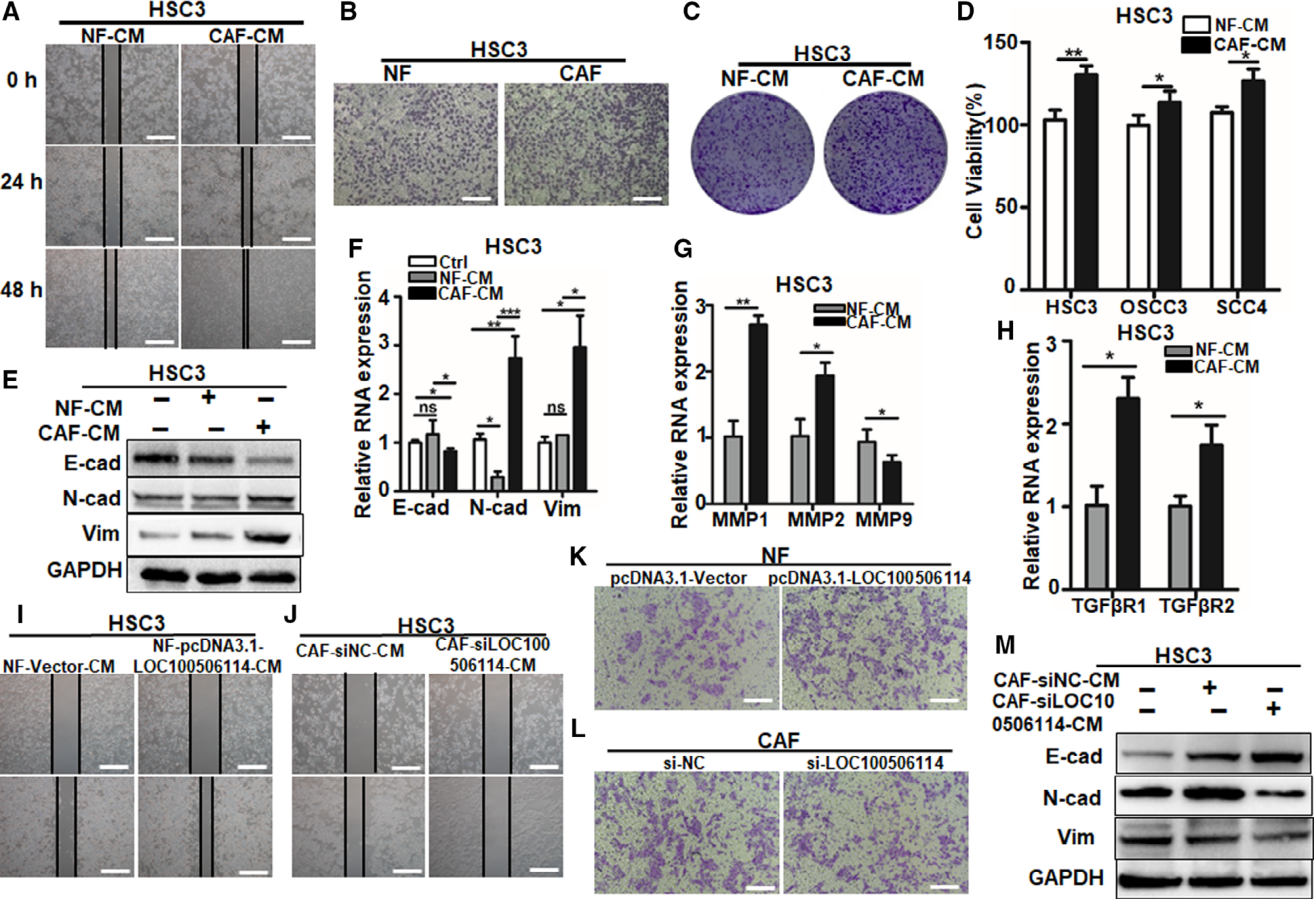
LOC100506114‐expressed CAF promote OSCC cell migration and proliferation. (A) The wound scratch assays of HSC3 treated with NF‐CM or CAF‐CM for 0, 24 and 48 h. Scale bars: 100 μm. (B) The migration of HSC3 was performed using Transwell assays. Scale bars: 100 μm. (C) The clone formation assays of HSC3 treated with NF‐CM or CAF‐CM. (D) The cell viability was detected by CCK‐8. (E) Western blots showing protein levels in HSC3 treated with NF‐CM or CAF‐CM for 48 h. (F) The expression of E‐cadherin, N‐cadherin and vimentin in HSC3 treated with NF‐CM or CAF‐CM for 48 h. (G) The expression of MMP1, MMP2 and MMP9 in HSC3 treated with NF‐CM or CAF‐CM for 48 h. (H) The expression of TGFβR1 and TGFβR2 in HSC3 treated with NF‐CM or CAF‐CM for 48 h. (I,J) The wound scratch assays of HSC3 treated for 24 h with conditioned medium from Lv‐LOC100506114 NF or Lv‐si‐LOC100506114 CAF. Scale bars: 100 μm. (K,L) Transwell migration assay showing the cell migration of HSC3. Scale bars: 100 μm. (M) The EMT transformation was reversed by CM of CAF transfected by lentivirus si‐LOC100506114. All data are presented as mean ± SD. *P < 0.05, **P < 0.01, ***P < 0.001. Representative data from three independent experiments.

To demonstrate that the expression of LOC100506114 in fibroblasts promotes the growth and metastasis of OSCC, we first treated OSCC cell lines HSC3, OSCC3 and SCC4 with the collected conditioned medium of NF, pcDNA3.1‐LOC100506114‐NF‐CM, CAF and si‐LOC100506114‐CAF‐CM, respectively. The wound scratch assay showed that the migration of the OSCC cell line was promoted more by CAF‐CM than by NF‐CM (Figs 3A and S2A,B). Consistent with the results of the wound scratch assay, we observed that CAF enhanced the migration of cancer cells compared with the group of NF co‐cultured with OSCC cell lines by the Transwell chamber (Figs 3B and S2C,D). CAF‐CM significantly promoted cell clone formation (Figs 3C and S2C,D) and increased the proliferation viability of OSCC cell lines (Fig. 3D). The metastasis of tumors is often associated with the epithelial–mesenchymal transition (EMT) in epithelial cells. To investigate the change in EMT of OSCC cell lines treated with NF‐CM or CAF‐CM, we examined the expression of epithelial cell markers E‐cadherin and stromal cell markers N‐cadherin and vimentin by western blot and qPCR. The results showed that the expression of E‐cadherin was downregulated and the expression of N‐cadherin and vimentin upregulated in HSC3, OSCC3 and SCC4 treated with CAF‐CM compared with the NF‐CM treatment group (Figs 3E,F and S2E–H). Furthermore, HSC3 cells treated with CAF‐CM expressed more MMP1 and MMP2 compared with the groups treated with NF‐CM (Fig. 3G); the same results occurred in OSCC3 and SCC4 cells treated with CAF‐CM (Fig. S2I,J). These results indicate that CAF promote EMT in tumor cells and cell migration. Additionally, we found that the expression of TGFβR1 and TGFβR2 was upregulated in HSC3, OSCC3 and SCC4 with CAF‐CM (Figs 3H and S2K,L). Moreover, the NF‐CM from LOC100506114‐overexpressed NF and enhanced HSC3 cell migration (Fig. 3I,K), whereas the CAF‐CM from CAF infected with the lentivirus si‐LOC100506114, impaired the cell migration of HSC3 (Fig. 3J,L). The same results occurred in the other two OSCC cell lines, OSCC3 and SCC4 (Fig. S2M,O and N, P). Additionally, the EMT was reversed in HSC3 treated with CM of CAF stably expressed si‐LOC100506114 by lentivirus (Fig. 3M). These results illustrate that the expression of LOC100506114 in CAF plays a critical role in the cell migration of tumor cells.

### 3.4 LOC100506114‐reprogrammed fibroblasts promote OSCC tumor growth *in vivo*

**Fig. 4:**
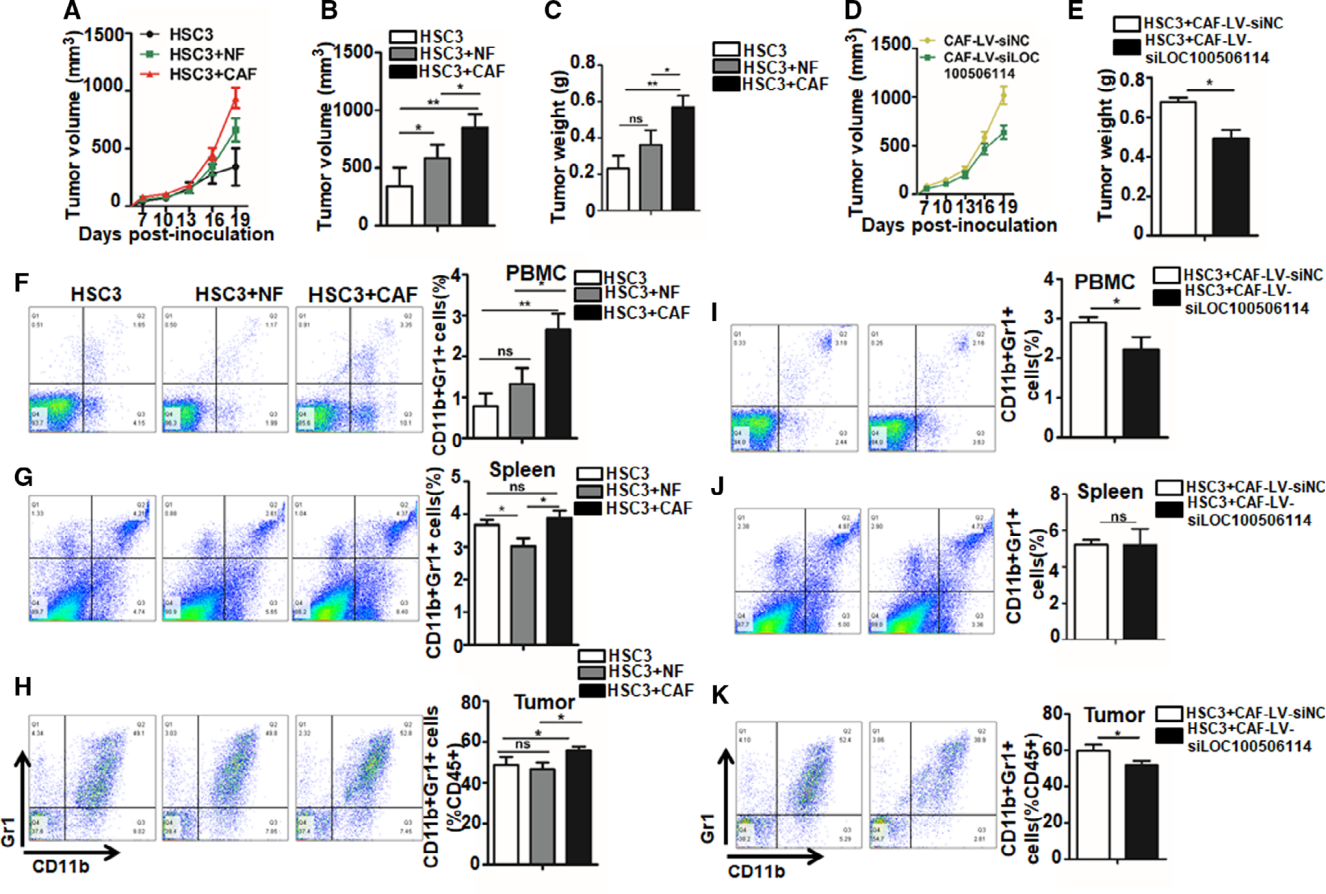
LOC100506114‐expressed CAF promote tumor formation and recruit MDSC *in vivo*. (A–C) CAF promote tumorigenicity in OSCC cancer xenograft models. HSC3 (1 × 10^6^ cells, *n* = 6) was injected subcutaneously with NF (0.5 × 10^6^ cells, *n* = 6) or CAF (0.5 × 10^6^ cells, *n* = 6). Tumor volume on the 19th day after subcutaneous injection (B) and tumor weight (C). (D,E) A xenograft model was established. HSC3 (1 × 10^6^ cells) was subcutaneously injected with CAF infected with the control lentiviral construction (0.5 × 10^6^ cells, *n* = 7) or with the lentiviral construction of stably expressed si‐LOC100506114 (0.5 × 10^6^ cells, *n* = 7), and tumor volume changes were recorded. Tumor volume on the 19th day after subcutaneous injection (D) and tumor weight (E). (F–H) The proportion of MDSC in PBMC, spleens and tumor in OSCC xenograft models by subcutaneous injection of HSC3 alone or co‐injection of HSC3 with NF or CAF. (I–K) The proportion of MDSC in PBMC, spleens and tumor in OSCC xenograft models by subcutaneous co‐injection of HSC3 with CAF infected with the control lentiviral construction or CAF infected with the lentiviral construction of stably expressed si‐LOC100506114. All data are presented as mean ± SD. Significance calculated using the unpaired *t*‐test. *P < 0.05, **P < 0.01.

To confirm that the LOC100506114‐expressing stromal fibroblasts exert a pro‐tumorigenic role in OSCC tumor *in vivo*, we constructed nude mouse xenograft models by respectively injecting subcutaneously HSC3 alone, with NF or with CAF. The tumor volume of co‐implanting HSC3 with CAF is obviously larger than another two groups. The results showed that CAF play a significant pro‐tumorigenic role compared with co‐injection with NF or HSC3 alone (Fig. 4A–C). Additionally, we separately detected the MDSC in PBMC, spleens and tumors of xenograft models. The analysis of flow cytometry showed that CAF significantly increased the proportion of MDSC in PBMC, spleens and tumors (Fig. 4F–H). To determine further the effect of LOC100506114 in CAF on pro‐tumorigenicity *in vivo*, we subcutaneously co‐injected HSC3 and CAF stably expressing si‐LOC100506114 or a control sequence into female nude mice. Compared with co‐transplant with the control CAF, those expressing si‐LOC100506114 significantly impaired tumor growth (Fig. 4D,E), as well as markedly decreasing the proportion of MDSC in PBMC and tumor (Fig. 4I–K). Collectively, the results support that LOC100506114‐expressed CAF contributes to promotion of tumor growth and the recruitment of MDSC.

### 3.5 LOC100506114 positively correlates with GDF10 in CAF from OSCC tumor patients and xenograft models

**Fig. 5:**
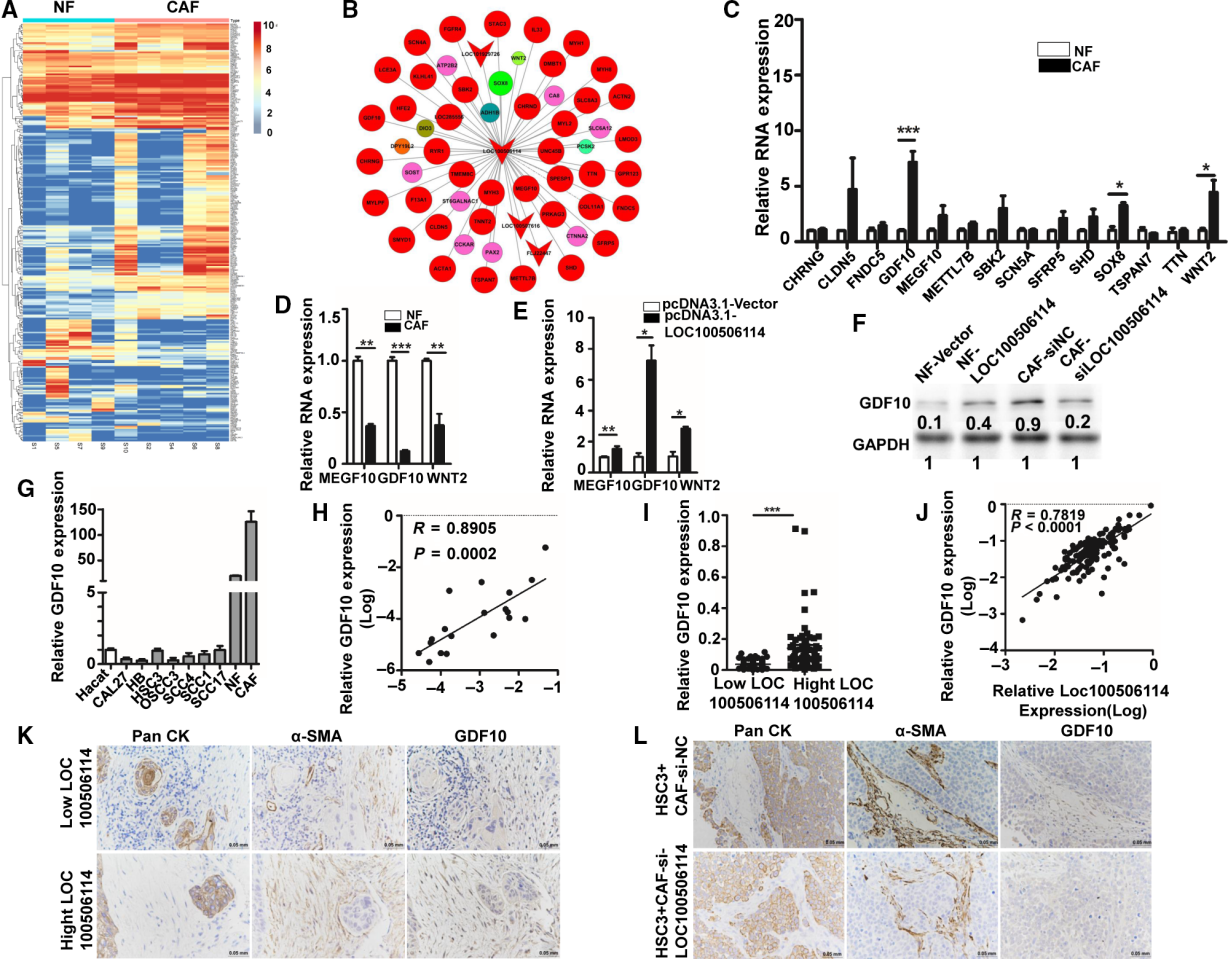
LOC100506114 positively correlates with GDF10 expression in CAF. (A) The heat map of protein‐coding genes differentially expresses NF and CAF. The expression of GDF10, MEGF10, WNT2 and SOX8 was higher in CAF than in NF (B) Analysis of the co‐expression network of LOC100506114. (C) The qPCR showing the expression of differentially expressed genes in the network map in NF and CAF. (D) The expression of GDF10, MEGF10 and WNT2 in CAF transfected with si‐NC or si‐LOC100506114. (E) The expression of GDF10, MEGF10 and WNT2 in NF transfected with pcDNA3.1‐Vector or pcDNA3.1‐LOC100506114. (F) Western blots showing the expression of GDF in CAF transfected with si‐NC or si‐LOC100506114 and NF transfected with pcDNA3.1‐Vector or pcDNA3.1‐LOC100506114. (G) The expression of GDF10 in human normal epithelial cells HaCaT, OSCC cells CAL27, HB, HSC3, OSCC3, SCC4, SCC1 and SCC17b, and NF and CAF. (H) Analysis of the correlation of LOC100506114 and GDF10 expression in 10 NF and 10 CAF. (I) The expression of GDF10 in OSCC patient tumor tissues with a high or low level of LOC100506114 (*n* = 140). (J) Correlation analysis of LOC100506114 and GDF10 expression in tumor tissues of OSCC patients (*n* = 140). (K) Representative IHC images showing PanCK, α‐SMA and GDF10 staining in OSCC patient tissue samples with a high or low level of LOC100506114. (L) Representative IHC images showing PanCK, α‐SMA and GDF10 staining in tumor samples of xenograft models. Scale bars: 50 μm. All data are presented as mean ± SD. Significance calculated using the unpaired *t*‐test. *P < 0.05, ** *P* < 0.01, ***P < 0.001. Representative data from three independent experiments.

To investigate further how LOC100506114 regulates the function of CAF, we performed gene co‐expression analysis of RNA‐seq data. We screened some genes that were significantly co‐expressed with LOC00506114 (Fig. 5A,B). The expression of GDF10, MEGF10, SOX8 and WNT2 in CAF was significantly higher than that of NF by qPCR (Fig. 5C). Finally, we determined the most significant expression of GDF10 between NF and CAF by knocking down LOC100506114 in CAF and overexpressing LOC100506114 in NF (Fig. 5D–F). These results indicate that LOC100506114 regulates the expression of GDF10 in CAF.

It is well known that GDF10 is usually suppressed in tumor cells. We detected the expression of GDF10 in NF and CAF, OSCC cell lines CAL27, HB, HSC3, OSCC3, SCC4, SCC1 and SCC17b, and human normal keratinocytes HaCaT by qPCR. The results showed that the expression of GDF10 in CAF was much higher than that of other cells, and the expression of GDF10 in some tumor cell lines (such as CAL27 and HB) was lower than that of normal epithelial cells (Fig. 5G). The expression of GDF10 was positively correlated with LOC100506114 in 10 NF and 10 CAF (Fig. 5H). To examine this correlation further, we detected the expression of GDF10 and LOC100506114 in 140 OSCC patient tissue samples. The expression level of GDF10 in tumor tissues of OSCC patients with a high expression of LOC100506114 was significantly higher than that of OSCC patients with a low expression of LOC100506114 (Fig. 5I). The correlation analysis further confirmed that the expression of GDF10 was positively correlated with LOC100506114 (Fig. 5J). It was concluded that the higher expression of GDF10 was at a high level in both the OSCC tumor sample and xenograft models co‐injected with HSC3, with CAF stably expressed by si‐NC, according to immunohistochemistry (Fig. 5K,L).

### 3.6 LOC100506114 elevates the expression of GDF10 by binding to RUNX2

**Fig. 6:**
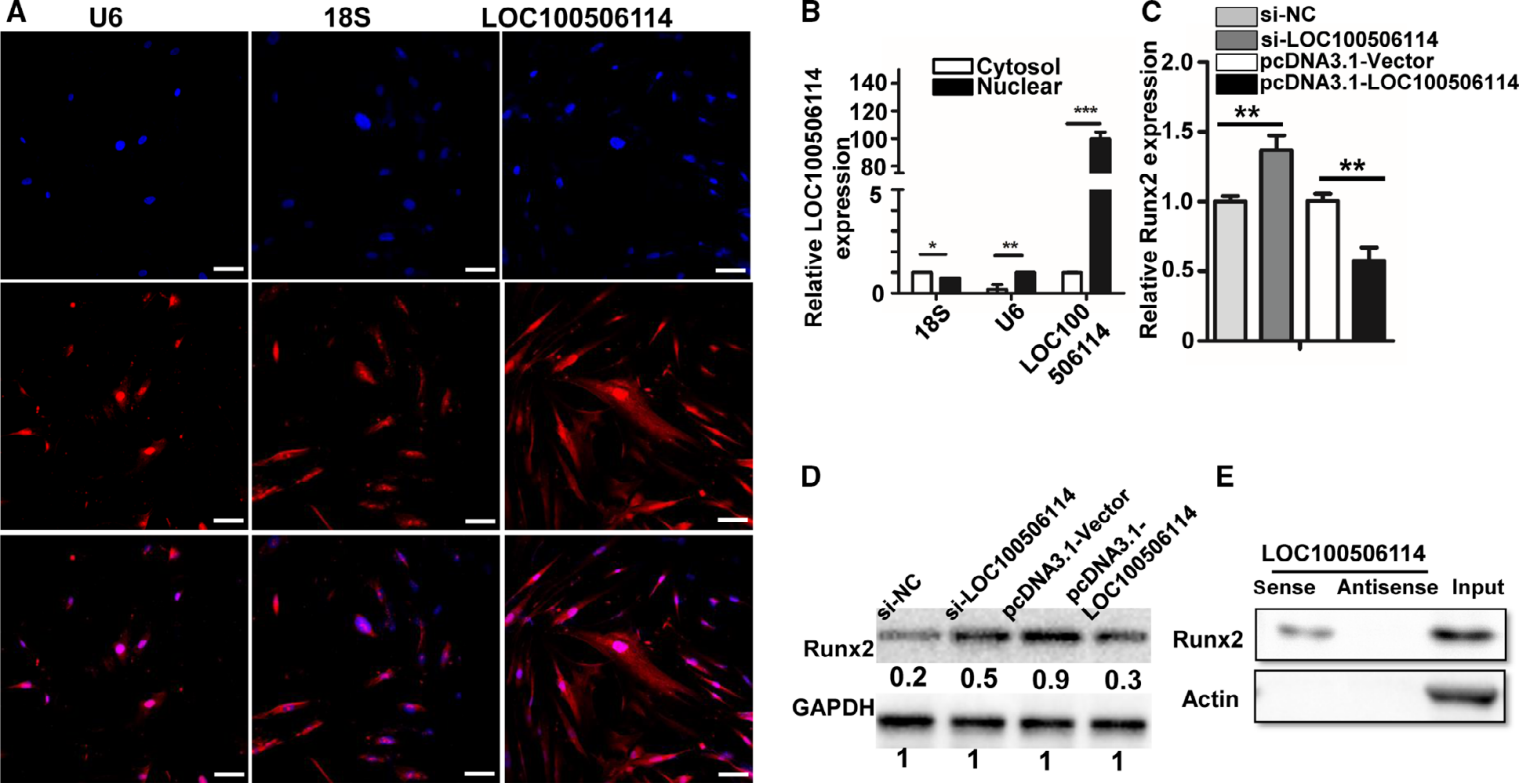
LOC100506114 upregulates the expression of GDF10 by binding to RUNX2. (A) FISH showing LOC100506114 expression mainly in the nucleus of CAF. Scale bars: 100 μm. (B) Separation of cytoplasm and nucleus. 18S was used as cytoplasm control and U6 as nuclear control. (C,D) The expression of RUNX2 in NF transfected with pcDNA3.1‐LOC100506114 and CAF transfected with si‐LOC100506114. (E) RNA binding protein assay; the antisense strand of LOC100506114 was used as negative control. All data are presented as mean ± SD. Significance calculated using the unpaired *t*‐test. *P < 0.05, **P < 0.01, ***P < 0.001. Representative data from three independent experiments.

Emerging evidence has shown that lncRNA can regulate gene expression by binding to protein or RNA [31]. To testify how LOC100506114 regulates GDF10 expression, we first detected its expression at the subcellular level using RNA FISH. Compared with the nucleus control U6 and the cytoplasm control 18S, LOC100506114 was mainly expressed in the nucleus of CAF (Fig. 6A). To confirm the expression of LOC100506114 at the subcellular level, we separated the cytoplasm and nucleus. The qPCR showed that LOC100506114 was mainly expressed in the nucleus (Fig. 6B). Moreover, a previous study showed that the transcription factor RUNX2 is able to bind to the promoter region of GDF10 to inhibit the expression of GDF10 [32]. Our results also showed that LOC100506114 affected the expression of RUNX2 (Fig. 6C,D). Therefore, we hypothesized that LOC100506114 regulates the expression of GDF10 by binding to the transcription factor RUNX2. RNA binding experiments also validated that the lncRNA LOC100506114 sense strand, rather than the antisense strand of lncRNA LOC100506114, binds to the transcription factor RUNX2 (Fig. 6E). These results indicate that the expression of GDF10 is upregulated by binding of LOC100506114 to RUNX2 in CAF.

### 3.7 CAF promote tumor cell proliferation and migration by LOC100506114‐mediated GDF10 secretion

**Fig. 7:**
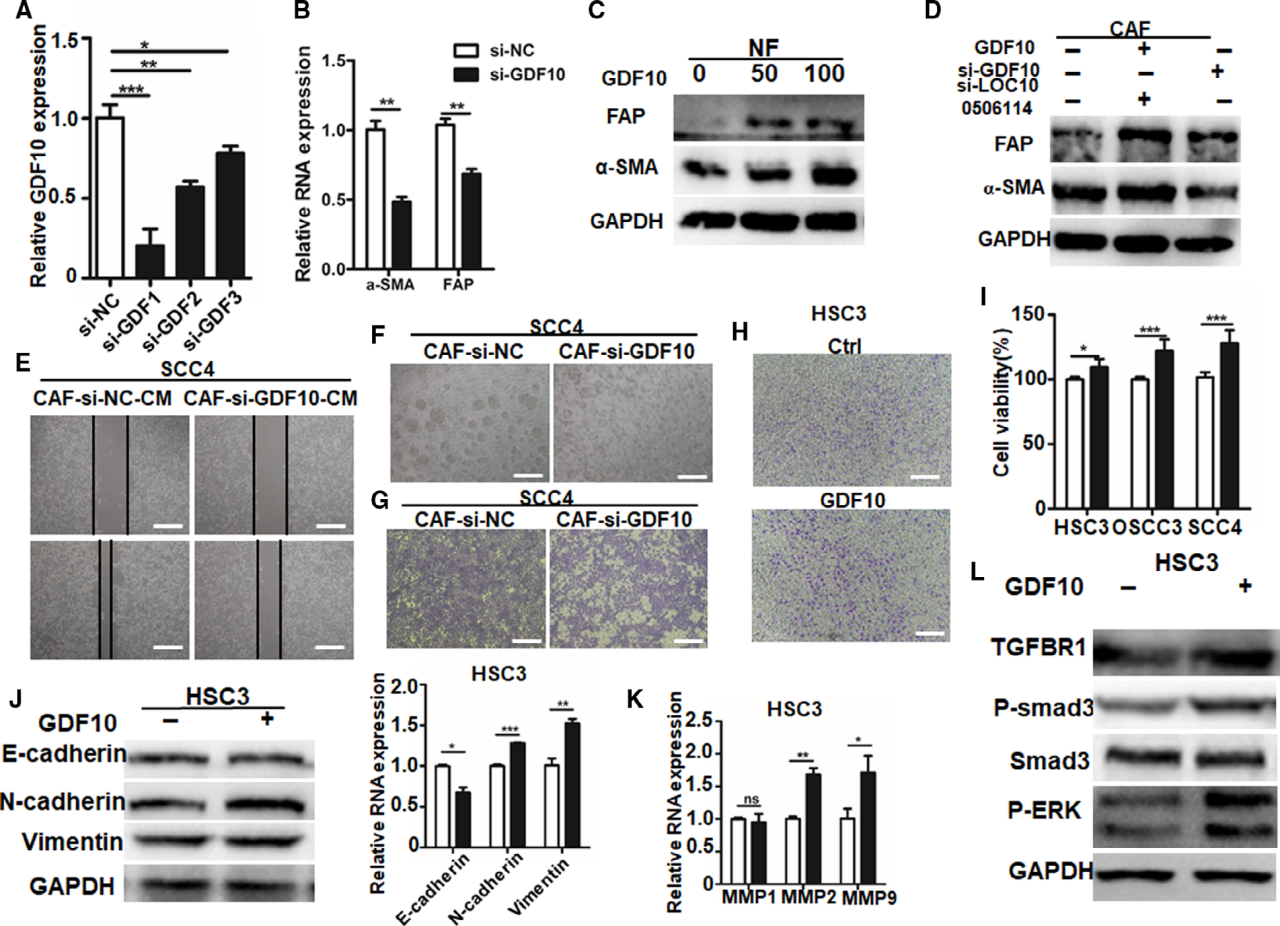
CAF regulate the expression of α‐SMA and FAP and promote the proliferation and migration of tumor cells by GDF10. (A) The expression of GDF10 in CAF transfected with si‐NC or si‐GDF10. Scale bar: 50 μm (B) The expression of α‐SMA and FAP in CAF transfected with si‐NC or si‐GDF10. (C) Western blots showing α‐SMA and FAP expression in NF treated with 0, 50 or 100 ng·mL^−1^ GDF10. (D) Western blots showing α‐SMA and FAP expression in CAF transfected with si‐N or si‐GDF10, or treated with 50 ng·mL^−1^ GDF10 after transfection with si‐LOC100506114. (E) Wound scratch assay showing the cell migration of SCC4 treated with CM from CAF transfected with si‐NC or si‐GDF10. Scale bars: 100 μm. (F) The cell proliferation of SCC4 co‐cultured with CAF transfected with si‐NC or si‐GDF10 by Transwell assay coated with thick Matrigel. Scale bars: 100 μm. (G) Transwell assay showing the migration of SCC4 co‐cultured with CAF transfected with si‐NC or si‐GDF10. Scale bars: 100 μm. (H) Transwell assay showing the migration of HSC3 treated with GDF10 (50 ng·mL^−1^) for 48 h. Scale bars:  100 μm. (I) The cell proliferation activity of HSC3, OSCC3 and SCC4 was detected by CCK‐8 assay after treatment with 50 ng·mL^−1^ GDF10 for 48 h. (J) Western blots showing protein levels in HSC3 treated with 50 ng·mL^−1^ GDF10 for 48 h. (K) MMP1, MMP2 and MMP9 expression in HSC3 treated with 50 ng·mL^−1^ GDF10 for 24 h. (L) Western blots showing protein levels in HSC3 48 h after treatment with 50 ng·mL^−1^ GDF10. All data are presented as mean ± SD. *P < 0.05, **P < 0.01, ***P < 0.001. Representative data from three independent experiments.

To verify how the effect of GDF10 on the transformation of CAF and tumor cell growth, we determined the expression of α‐SMA and FAP in CAF transfected with si‐GDF10 by qPCR. Compared with the negative control group, the downregulated expression of GDF10 significantly reduced the expression of α‐SMA and FAP in CAF (Fig. 7A,B). Western blots showed that the expression of α‐SMA and FAP was significantly upregulated in NF treated with exogenous recombinant human GDF10 (Fig. 7C). Conversely, the expression of α‐SMA and FAP was decreased in CAF transfected with si‐GDF10 but was restored in CAF transfected with si‐LOC100506114 and the addition of 50 ng·mL^−1^ GDF10 (Fig. 7D). These results suggest that GDF10 promotes the transformation of NF into CAF.

To demonstrate that LOC100506114‐reprogrammed CAF, promoted tumor cell migration and growth by secreting GDF10, we treated OSCC cell line SCC4 with the CM from CAF transfected with si‐GDF10 or si‐NC. In the wound scratch assay and Transwell migration assay, CM from CAF transfected with si‐GDF10 significantly inhibited the migration of SCC4 (Fig. 7E,G). Co‐culture with si‐GDF10‐CAF, by coating the Transwell chamber with the thick Matrigel, significantly inhibited the proliferation of SCC4 compared with CAF transfected with si‐NC (Fig. 7F). To demonstrate further that the expression of GDF10 in CAF promotes tumor cell migration, we added tumor cells with exogenous human recombined GDF10. The Transwell migration showed that GDF10 promoted cell migration (Figs 7H and S3A,B), as well as the proliferation of HSC3, OSCC3 and SCC4 (Fig. 7I). Furthermore, compared with the control groups, the expression of E‐cadherin was downregulated and the expression of N‐cadherin and vimentin upregulated in GDC10‐treated HSC3, OSCC3 and SCC4 at protein level (Figs 7J and S3C,D). Consistent with the results of protein expression, the expression of E‐cadherin was downregulated and the expression of N‐cadherin and vimentin upregulated in GDC10‐treated HSC3, OSCC3 and SCC4 at RNA level (Figs 7J and S2C,D). In addition, MMP1, MMP2 and MMP9, which are closely related to tumor cell migration, were significantly upregulated in the GDF10‐treated group (Figs 7K and S2E,F). Finally, the expression of TGFβR1 in GDF10‐treated tumor cells was upregulated (Fig. 7L), which is consistent with the upregulation of TGFβR1 expression in the treatment of CAF‐CM. The Smad3 associated with the transforming growth factor‐β (TGFβ) signaling pathway was significantly activated and ERK phosphorylation levels increased (Fig. 7L). These results illustrated that CAF promote cell proliferation and migration by GDF10‐mediated activation of the TGFβR1/Smad3/ERK pathway in tumor cells.

## 4 Discussion

Although the treatment of cancer, including OSCC, has developed rapidly in recent years, the high recurrence and metastasis rate is still a problem that threatens the survival of OSCC patients. There is increasing evidence that CAF play an important role in the growth and metastasis of many types of tumor [33, 34, 35]. Therefore, understanding the mechanisms of interaction between CAF and tumor cells may contribute to the future development of OSCC therapy. As CAF have not yet been identified by specific cell surface markers, there are a large number of reports that CAF‐expressing biological markers such as α‐SMA, FAP, FSP‐1 and vimentin can distinguish CAF by combining several markers with cell morphology [6, 34]. We identified the CAF by immunofluorescence detection of FAP and α‐SMA in the expression of interstitial fibroblasts isolated from OSCC patients and in combination with the cell morphology exhibited by CAF under light microscopy. Consistent with previous studies, CAF expressed higher levels of FAP and α‐SMA compared with NF in our study. In the past, lncRNA has been considered to be the ‘noise’ of genomic transcription but, recently, more and more evidence has shown that lncRNA is involved in many biological processes, such as tissue development and cell differentiation [31, 36], and in many diseases, functionally regulating the gene through various mechanisms [37, 38]. In this study, we screened a new, undescribed lncRNA, LOC100506114, by analyzing the differential expression of lncRNA genes in NF and CAF. In terms of interstitial fibroblast reprogramming, many non‐coding RNA are involved. Mitra *et al*. [39] have shown that reprogramming fibroblasts to become CAF through the action of miRNA, promotes tumor growth and progress. Melling *et al*. [40] have also demonstrated that miR‐145 targets the TGFβ signaling pathway to form a negative feedback loop, and promotes primary human fibroblasts to obtain a heterogeneous phenotype of CAF that supports tumor cell invasion and metastasis. In addition, previous studies in our laboratory have shown that lncRNA‐CAF is involved in the functional transformation of primary normal human fibroblasts to CAF [26]. In this study, we further demonstrated the role of lncRNA in the reprogramming of mesenchymal fibroblasts.

Based on bioinformatics analysis of RNA‐seq data to obtain a co‐expression network, we found that LOC100506114, which is upregulated in CAF, is directly related to growth factor GDF0. As a member of the TGFβ superfamily, GDF10 is also known as BMP3B because of its similarity to BMP3. Emerging evidence shows that GDF10 promotes the differentiation and growth of osteoblasts and plays a role in fracture healing, strokes and various tumors [41]. A recent study by Yu‐Lee *et al*. [42] suggests that GDF10 induces dormancy of prostate cancer in combination with TGFβ2 by means of the activation of TGFβRIII‐p38MAPK‐pS249/pT252‐RB signaling. In addition, Geet *et al*. [43] demonstrated that their selective activation of TGFβR‐dependent Smad3 phosphorylation is involved in breast cancer. In the present study, we found that GDF10 also promotes the growth and migration of OSCC tumor cells by activating the TGFβRI/Smad3/ERK pathway. In addition, we demonstrated that GDF10 promotes the functional transformation of normal human NF into CAF by LOC100506114 binding to transcription factor RUNX2, to support tumor cell growth, invasion and metastasis.

## 5 Conclusions

In summary, our current research provides new and powerful evidence for tumor stromal cells affecting tumorigenesis and progression. We have revealed the role of lncRNA LOC100506114 in the functional transition of NF to CAF, and the proliferation and migration of OSCC cells by activating the TGFβR1/Smad3/ERK pathway. Our research provides a potential focus for cancer treatment strategies in the future.

## Conflict of interest

The authors declare no conflict of interest.

### Peer Review

The peer review history for this article is available at https://publons.com/publon/10.1002/1878‐0261.12935.

## Author contributions

D.Z., Y.S. and X.L. designed the experiments. D.Z. operated the experiments. D.Z., L.D., G.S. and L.D. carried out data analysis and interpretation. L.D. provided the method of isolating CAF from tumor tissue. D.Z. and Y.S. wrote and revised the paper. Y.Y.H. provided the concept and design of this study. Y.W., Y.N. and Y.H. co‐designed experiments and co‐wrote the manuscript. All authors reviewed the manuscript.

## Ethics approval and consent to participate

Ethics involving patient’s samples of this study were approved by the Research Ethics Committee of Nanjing Stomatology Hospital Affiliated to Nanjing University and were carried out in accordance with the approved guidelines. Informed consent was obtained from all subjects enrolled in the studies who provided the samples. All specimens were handled and anonymized according to ethical and legal standards. Procedures involving animal experiments were approved by the Institutional Committee on Animal Care, Nanjing University.

## Supporting information


**Fig. S1.** The characteristics of CAFs in OSCC.
**Fig. S2.** LOC100506114‐reprogrammed CAFs promote OSCC cell migration and proliferation.
**Fig. S3.** GDF10 promotes tumor cell proliferation and migration.
**Fig. S4.** The heat map provides a visual representation of the differentially expressed RNA between NF and CAF.
**Table S1.** Primers used for real‐time quantitative PCR.
**Table S2.** The sequence of RNAi for LOC100506114 and GDF10.

## Data Availability

All data generated or analyzed during this study are included either in this article or in the supplementary Materials and Methods, Tables, Figures, and Figure Legends files. The data supporting the findings of this study are available from the corresponding author (yayihou@nju.edu.cn) upon reasonable request.
