# Supplementary material for: Cancer‐associated fibroblasts promote tumor progression by lncRNA‐mediated RUNX2/GDF10 signaling in oral squamous cell carcinoma
Source: Mol Oncol. 2021 Jun 10;16(3):780–94. doi: 10.1002/1878-0261.12935 (PMC8807363; doi:10.1002/1878-0261.12935)
Supplement: Supplementary file 1 — Fig. S1. The characteristics of CAFs in OSCC. Fig. S2. LOC100506114‐reprogrammed CAFs promote OSCC cell migration and proliferation. Fig. S3. GDF10 promotes tumor cell proliferation and migration. Fig. S4. The heat map provides a visual representation of the differentially expressed RNA between NF and CAF. Table S1. Primers used for real‐time quantitative PCR. Table S2. The sequence of RNAi for LOC100506114 and GDF10. [file MOL2-16-780-s001.docx]

**Cancer-associated fibroblasts promote tumor progression by lncRNA-mediated RUNX2/GDF10 signaling in** **oral squamous cell carcinoma**

Zhang Dongya^1#^, Song Yuxian^2#^, Li Dan^1^, Liu Xinghan^1^, Pan Yuchen^1^, Ding Liang^2^, Shi Guoping^1^, Wang Yong^3,4^, Ni Yanhong^2*^, Hou Yayi^1,3*^

**^1^**The State Key Laboratory of Pharmaceutical Biotechnology, Division of Immunology, Medical School, Nanjing University, Nanjing 210093, China.

**^2^** Central laboratory of Stomatology, Nanjing Stomatological Hospital, Medical School of Nanjing University, Nanjing, 210008, China.

**^3^** Jiangsu Key laboratory of Molecular Medicine, Nanjing 210093, China

**^4^** State Key Laboratory of Analytacal Chemistry for Life Science, Medical School, Nanjing University, Nanjing 210093, China

**#** Contribute equally to this work

* Corresponding author: Yayi Hou, The State Key Laboratory of Pharmaceutical Biotechnology, Division of Immunology, Medical School, Nanjing University, No.22 Hankou Rd., Gulou District, Nanjing 210093, Jiangsu, China. Tel/Fax: +86-25-83686341; Email: yayihou@nju.edu.cn

Or Yanhong Ni, Central Laboratory, Hospital of Stomatology, Medical School, Nanjing University, Nanjing, 210008. Tel: +86-025-83620140. Email: niyanhong12@163.com

**Table S1. Primers used for real-time quantitative PCR**

| Gene | Sequence |
| --- | --- |
| Human LOC100506114  Human FAP  Human α-SMA  Human GDF10  Human CHRNG  Human SHD  Human METTL7B  Human SFRP5  Human TSPAN7  Human CLDN5  Human SBK2  Human MEGF10  Human SOX8  Human TTN  Human WNT2  Human Runx2  Human TGFβR1  Human TGFβR2  Human MMP1  Human MMP2  Human MMP9  Human GAPDH | Forward: ACCTTGCCTGAAGGACCAAC  Reverse: GGTTGCAGCTCCGTTCTTTG  Forward: ATGAGCTTCCTCGTCCAATTCA  Reverse: AGACCACCAGAGAGCATATTTTG  Forward: CCAAGCCCTCACCTACTTCC  Reverse: GGCAGTGTAACTCTTCTGCAT  Forward: GGACTTTGACGAGAAGACGATG  Reverse: TCTTAGGCATGGGGAACTCAC  Forward: TGGACGGTGTCTTCGAGGT  Reverse: GCGGCAGCCAGTAGATACA  Forward: CTACGGGACTACCTGAGCTTT  Reverse: CTCTCCGCGTCCTCATAGG  Forward: CCTGCCTAGACCCAAATCCC  Reverse: AAACCGCTCATATTGGAGGTG  Forward: GTGCTGCACATGAAGAATGGC  Reverse: GCCCCGTAGAAGAAAGGGT  Forward: ACCAAACCTGTGATAACCTGTCT  Reverse: AGGGAGATATAGGTGCCCAGA  Forward: CTCTGCTGGTTCGCCAACAT  Reverse: CAGCTCGTACTTCTGCGACA  Forward: ATGCCCGGCAAACAGTCTG  Reverse: CACTCAGCGTCATCATGTCCT  Forward: ACAGAGTCAGCTATCGGACAG  Reverse: TTTGGAGCAATACAGCGACCA  Forward: CAAGGGCTACGACTGGAGTCT  Reverse: CATGCGGCTTGGCTTTGAG  Forward: CCCCATCGCCCATAAGACAC  Reverse: CCACGTAGCCCTCTTGCTTC  Forward: GATGCGTGCCATTAGCCAG  Reverse: AGATTCCCGACTACTTCGGAG  Forward: CCGCCTCAGTGATTTAGGGC  Reverse: GGGTCTGTAATCTGACTCTGTCC  Forward: GCTGTATTGCAGACTTAGGACTG  Reverse: TTTTTGTTCCCACTCTGTGGTT  Forward: AAGATGACCGCTCTGACATCA  Reverse: CTTATAGACCTCAGCAAAGCGAC  Forward: GGGGCTTTGATGTACCCTAGC  Reverse: TGTCACACGCTTTTGGGGTTT  Forward: CCCACTGCGGTTTTCTCGAAT  Reverse: CAAAGGGGTATCCATCGCCAT  Forward: AGACCTGGGCAGATTCCAAAC  Reverse: CGGCAAGTCTTCCGAGTAGT  Forward: AGAAGGCTGGGGCTCATTTG  Reverse: AGGGGCCATCCACAGTCTTC |

**Table S2. The sequence of RNAi for LOC100506114 and GDF10**

| si-LOC100506114-1 | sense 5＇-GAAGACCAGCAACUGCUAATT-3＇  antisense 5＇-UUAGCAGUUGCUGGUCUUCTT-3＇ |
| --- | --- |
| si-LOC100506114-2 | sense 5＇-GCAAAGAACGGAGCUGCAATT-3＇  antisense 5＇-UUGCAGCUCCGUUCUUUGCTT-3＇ |
| si-LOC100506114-3 | sense 5＇- GAGGGAGAAGUUGUAAUAUTT -3＇  antisense 5＇- AUAUUACAACUUCUCCCUCTT -3＇ |
| si-GDF10-1 | sense 5＇- CCAUGCAAGACUCGGAAAUTT-3＇  antisense 5＇- AUUUCCGAGUCUUGCAUGGTT-3＇ |
| si-GDF10-2 | sense 5＇- GCUGGAAUGAAUGGAUAAUTT-3＇  antisense 5＇- AUUAUCCAUUCAUUCCAGCTT-3＇ |
| si-GDF10-3 | sense 5＇- CCUGCUGUGUUCCCGAUAATT-3＇  antisense 5＇- UUAUCGGGAACACAGCAGGTT-3＇ |

**Supplementary Figure 1**


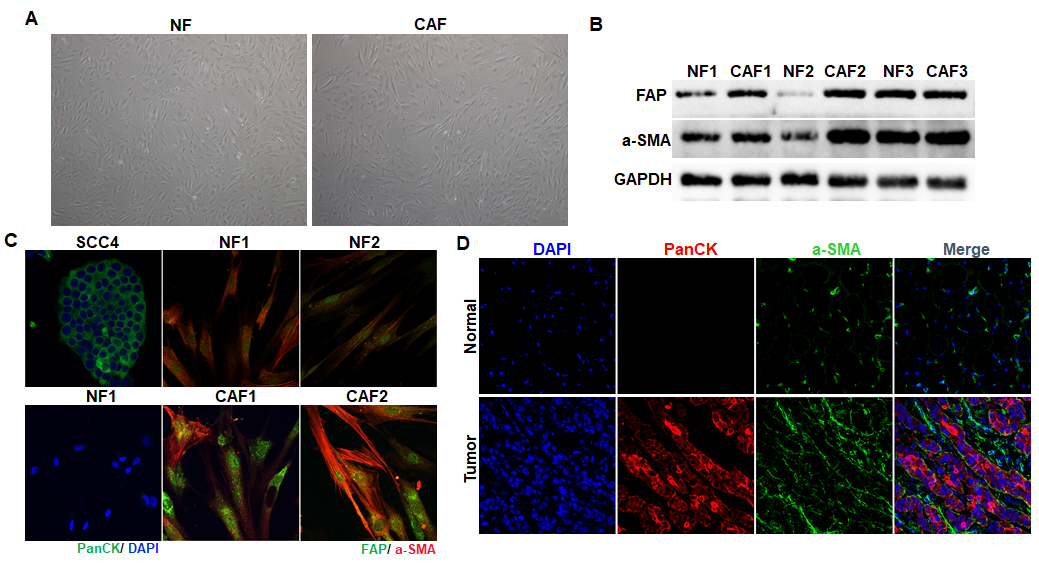


**Figure 1.** **The Characteristics of CAFs in OSCC** (A) The morphology of NFs and CAFs under the light microscopy. (B) The biomarker of CAFs-like phenotype express in NFs and CAFs at protein level. (C) The expression of FAP and α-SMA in NFs and CAFs were determined by immunofluorescence. (D) NFs and CAFs respectively in normal tissue and tumor tissue.

**Supplementary Figure 2**


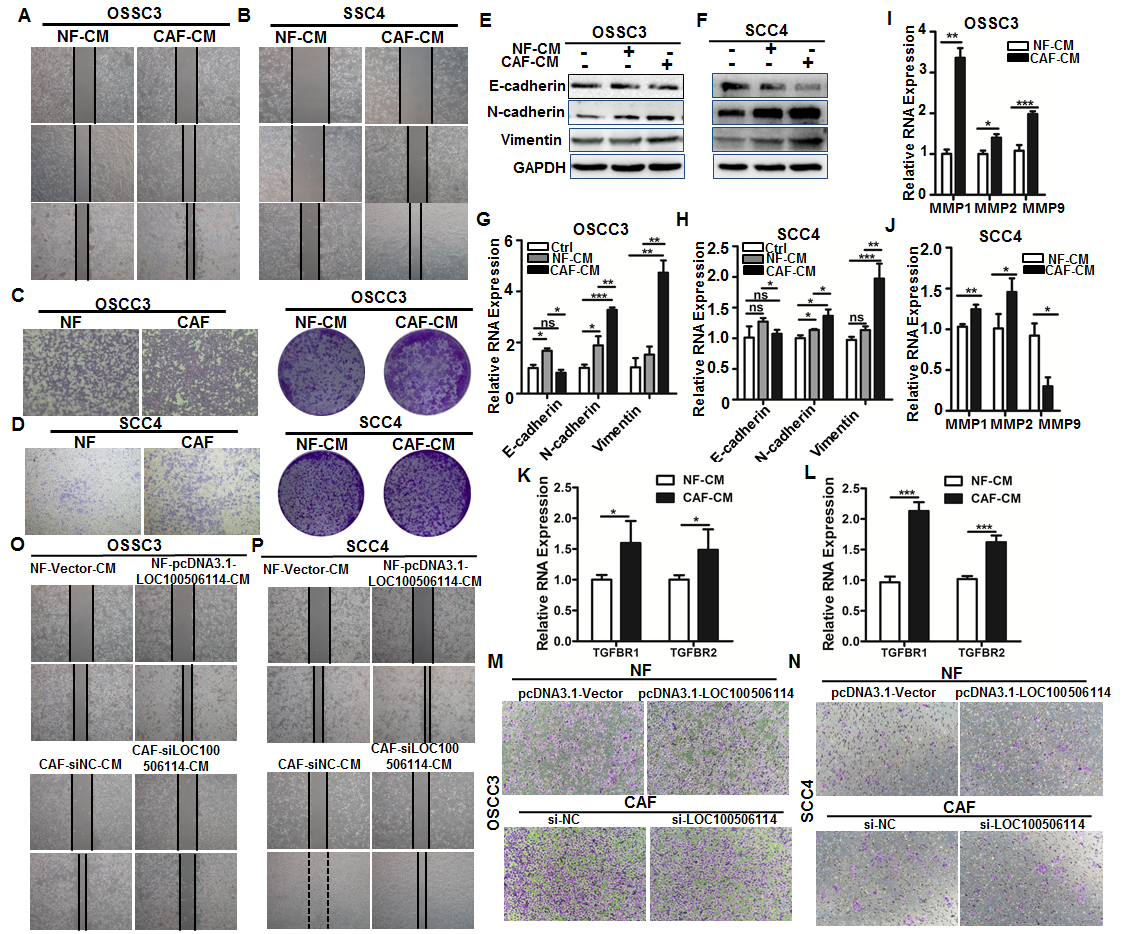


**Figure 2.** **LOC100506114-reprogrammed CAFs promote OSCC cell migration and proliferation.** (A) The wound scratch assays of HSC3 treated with NFs-CM and CAFs-CM for 0h, 24h and 48h, respectively, photographed at corresponding time. (B) The migration of HSC3 was performed by Transwell assay. The tumor cells were seeded in the upper chamber at 3x10^4^ cells/well, the lower chamber was planted with NFs or CAFs(8x10^4^ cells/well) for 48h.（C）The clone formation assays of HSC3 treated with NFs-CM or CAFs-CM. The cells were incubated 7 days with NFs-CM: medium for 1:1 or CAFs-CM: medium for 1:1 after seeding in 6-well plate(2x10^3^ cells/well). (D) The cell viability of HSC3, OSCC3 and SCC4 was detected by CCK-8. HSC3, OSCC3 and SCC4 were detected by CCK-8 before the treatment of NFs-CM or CAFs-CM for 48h after seeding in 96-well plate at 5x10^3^ cells/well for 24h. (E) The expression of E-cadherin, N-cadherin and Vimentin was determined by western blot in HSC3 treated with NFs-CM or CAFs-CM for 48h. (F) The expression of E-cadherin, N-cadherin and Vimentin in HSC3 treated with NFs-CM or CAFs-CM for 48h. (G)The expression of MMP1, MMP2 and MMP9 in HSC3 treated with NFs-CM or CAFs-CM for 48h. (H)The expression of TGFβR1 and TGFβR2 in HSC3 treated with NFs-CM or CAFs-CM for 48h. (I) The wound scratch assays of HSC3 treated with conditional medium from NFs infected with the lentivirus pcDNA3.1-LOC100506114. (J) The wound scratch assays of HSC3 treated with conditional medium from CAFs infected with the lentivirus piLenti-siRNA-LOC100506114. (K) The cell migration was performed by transwell assay for 48h. HSC3 cells were seeded in the upper chamber, and NFs infected with the lentivirus pcDNA3.1-LOC100506114 were seeded in the lower chamber. (L) The cell migration was performed by transwell assay for 48h. HSC3 cells were seeded in the upper chamber, and CAFs infected with the lentivirus piLenti-siRNA-LOC100506114 or piLenti-vector was seeded in the lower chamber. All data is mean±SD. Significance calculated using the unpaired t-test. *p<0.05, ** p<0.01, *** p<0.001. Representative data are from three independent experiments.

**Supplementary Figure 3**


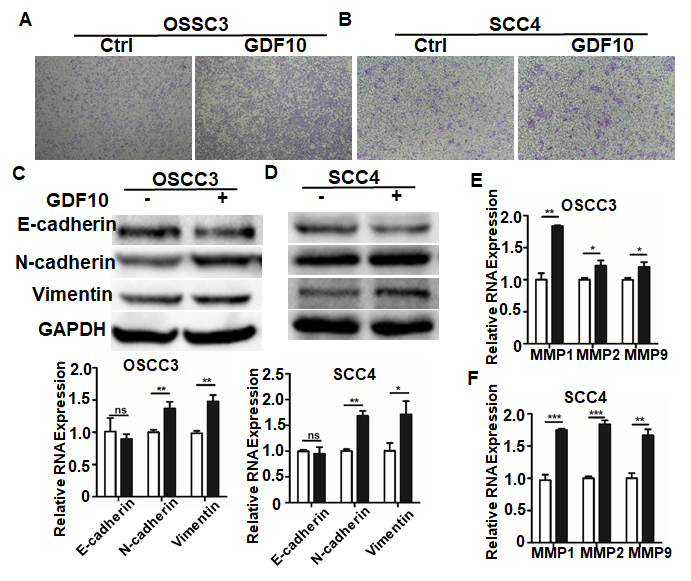


**Figure 3.** **GDF10 promotes tumor cell proliferation and migration** (A and B) The transwell assay showing the migration of OSCC3 and SCC4 treated with exogenous human recombinant GDF10(50ng/mL) for 48h. (C and D) The expression of E-cadherin, N-cadherin and Vimentin is determined by western blots and qPCR in OSCC3 and SCC4 treated with 50 ng/mL GDF10 for 48 h. (E and F) MMP1, MMP2 and MMP9 express in OSCC3 and SCC4 cells treated with 50 ng/mL GDF10 for 24 h. All data is mean±SD. Significance calculated using the unpaired t-test. *p<0.05, ** p<0.01, *** p<0.001. Representative data are from three independent experiments.


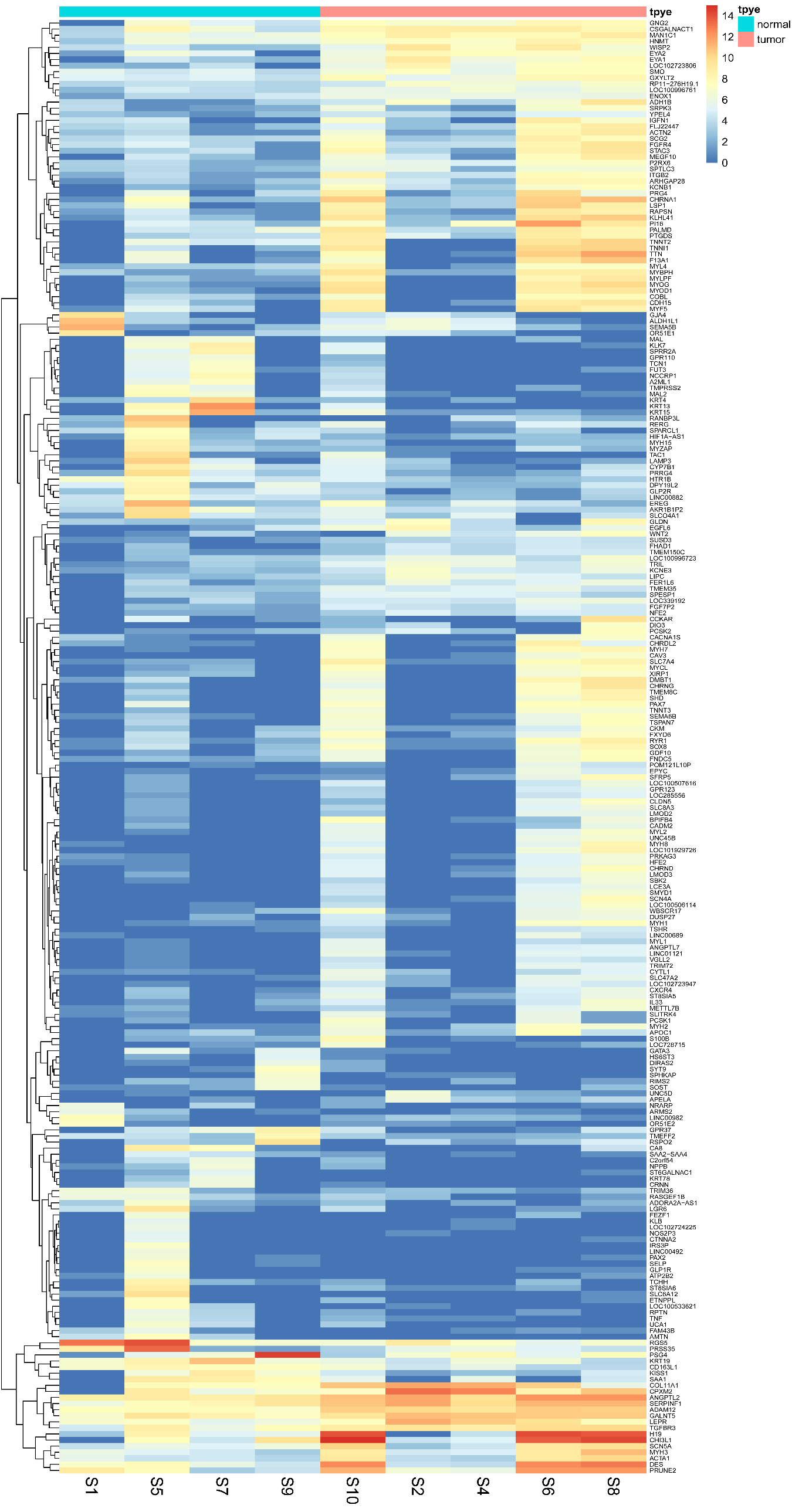
**Supplementary Figure 4**
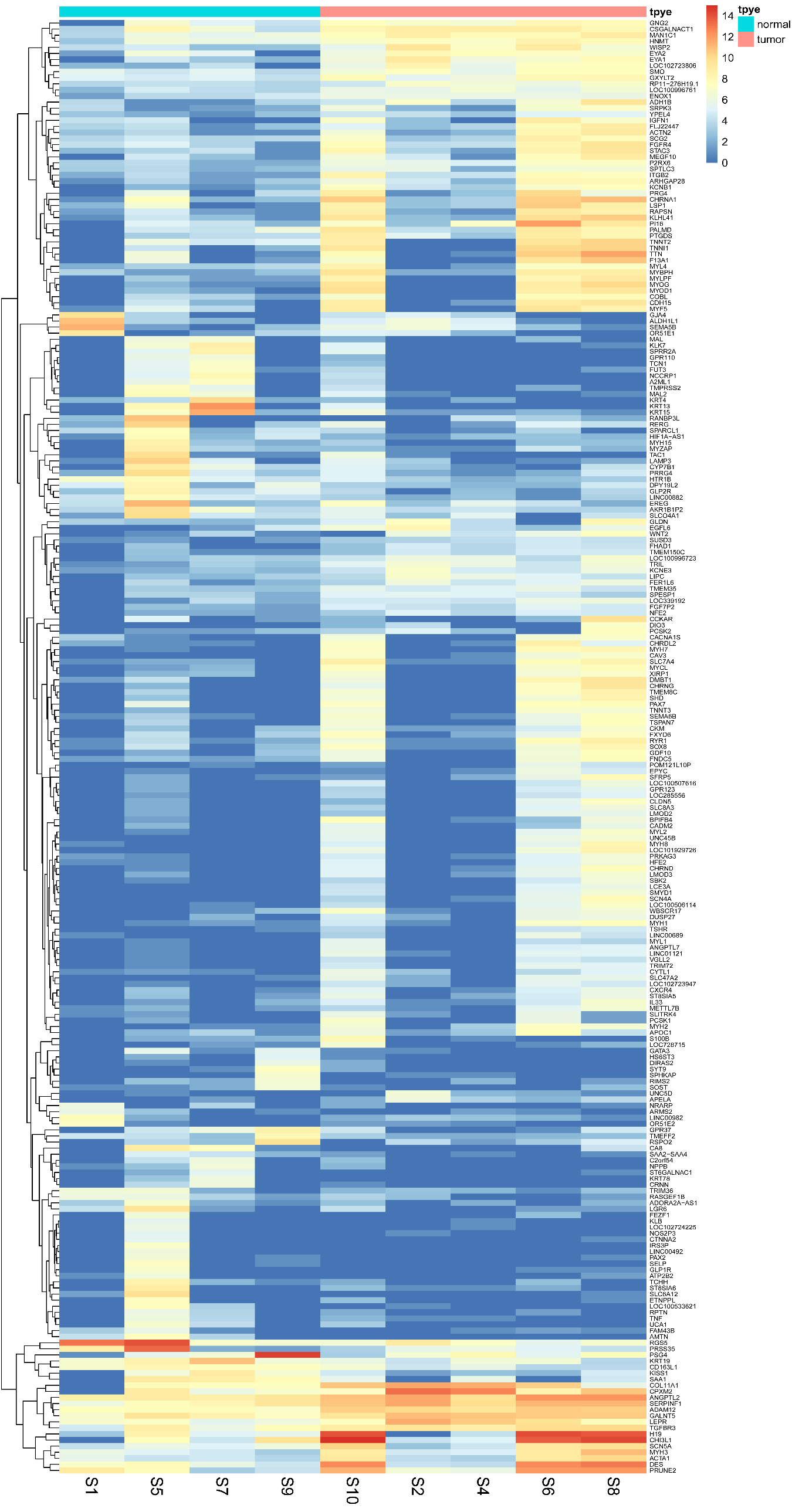


**Figure 4.** **The heat map provided a visual representation of the significant** **deregulated RNA between NFs and CAFs.** Screened with a fold change ratio of more than 2 times and a false discovery rate (FDR) of less than 0.05, (n=5 vs 5).
